# Control of Presynaptic Parallel Fiber Efficacy by Activity-Dependent Regulation of the Number of Occupied Release Sites

**DOI:** 10.3389/fnsys.2019.00030

**Published:** 2019-07-17

**Authors:** Hartmut Schmidt

**Affiliations:** Carl-Ludwig Institute for Physiology, Medical Faculty, University of Leipzig, Leipzig, Germany

**Keywords:** parallel fiber, faciliatation, replenishment, release probability, vesicle pools, residual calcium, synaptotagmin 7, Munc13

## Abstract

Parallel fiber (PF) synapses show pronounced and lasting facilitation during bursts of high-frequency activity. They typically connect to their target neurons *via* a single active zone (AZ), harboring few release sites (~2–8) with moderate initial vesicular release probability (~0.2–0.4). In light of these biophysical characteristics, it seems surprising that PF synapses can sustain facilitation during high-frequency periods of tens of action potentials (APs). Recent findings suggest an increase in the number of occupied release sites due to ultra-rapid (~180 s^−1^), Ca^2+^ dependent recruitment of synaptic vesicles (SVs) from replenishment sites as major presynaptic mechanism of this lasting facilitation. On the molecular level, Synaptotagmin 7 or Munc13s have been suggested to be involved in mediating facilitation at PF synapses. The recruitment of SVs from replenishment sites appears to be reversible on a slower time-scale, thereby, explaining that PF synapses rapidly depress and ultimately become silent during low-frequency activity. Hence, PF synapses show high-frequency facilitation (HFF) but low-frequency depression (LFD). This behavior is explained by regulation of the number of occupied release sites at the AZ by AP frequency.

## Introduction

Parallel fiber (PF) synapses are major sites for conveying sensory information to the cerebellar cortical output neurons, the Purkinje cells (PCs), and to interneurons. They are formed by granule cells, which fire bursts of action potentials (APs) over a broad range of frequencies up to ~1 kHz in response to sensory input (Chadderton et al., 2004; Rancz et al., 2007; Ritzau-Jost et al., 2014). PF synapses in turn are adapted to reliably respond to these high-frequency bursts of APs with sustained and facilitating transmission (Valera et al., 2012). This puts substantial demands on the mechanisms of synaptic vesicle (SV) supply.

Briefly, an AP invading a presynaptic terminal opens voltage-gated Ca^2+^ channels and the inflowing Ca^2+^ ions trigger the fusion of SVs with the presynaptic plasma membrane and transmitter release. Fusion of SVs is a probabilistic process that takes place at the presynaptic active zone (AZ). The AZ is thought to harbor one or more release sites (*N*) that constitute the individual entities at which a single SV can fuse with a certain vesicular release probability (*p*_v_; Südhof, 2013; Kaeser and Regehr, 2017).

For a single AP, the presynaptic efficacy depends on the number of release sites occupied by release-ready SVs (*N*_occ_) at the time of the AP and on the *p*_v_ of these SVs. The *p*_v_, in turn, depends on several factors, including the diffusional distance between the Ca^2+^ channels and the SV and the intrinsic Ca^2+^ sensitivity of its release machinery (Eggermann et al., 2012; Bornschein and Schmidt, 2019). While in experiments typically only an average *p*_v_ can be estimated (Clements and Silver, 2000), the *p*_v_ need not be homogeneous across release sites (Neher, 2015).

During a train of APs, the regulation of presynaptic efficacy gets more complex. Occupied release sites are continuously emptied by the fusion processes, which, without further mechanisms, would result in synaptic depression due to progressive depletion of the pool of release-ready SVs. How effectively the information transfer can be maintained during an AP train now depends on the speed with which *N*_occ_ can be restored or newly recruited and on their *p*_v_, which may increase. If the latter outcompetes SV consumption, the synapse may show facilitation rather than depression during the train (Jackman and Regehr, 2017; Neher and Brose, 2018).

This mini review article focusses on recent results from PF synapses suggesting that during high-frequency trains of APs the rate of restoration or recruitment of release sites exceeds the fusion rate, resulting in an activity-dependent increase in *N*_occ_ as major presynaptic mechanism of facilitation at PF synapses (Valera et al., 2012; Brachtendorf et al., 2015; Miki et al., 2016; Doussau et al., 2017).

## Parallel-Fiber Synapses

### Biophysics of Parallel Fiber Terminals

The target neurons of PFs include PCs and molecular layer interneurons (MLIs). PFs contact their targets typically by a single presynaptic bouton harboring a single AZ only (Xu-Friedman et al., 2001). Presynaptic Ca^2+^ transients are reliably induced by single APs, show very little trial-to-trial variability for a given bouton and linear summation during a train of APs (Brenowitz and Regehr, 2007; Schmidt et al., 2013; Baur et al., 2015; Miki et al., 2016; Kusch et al., 2018). Mature PF terminals gate release with P/Q-type channel nanodomains (Schmidt et al., 2013; Kusch et al., 2018) that develop from P/Q- and N-type channel microdomains gating release from young terminals (Mintz et al., 1995; Baur et al., 2015). Depending on their target neuron, PFs release SVs with *p*_v_ ~0.25–0.4 in 2 mM extracellular Ca^2+^ concentration ([Ca^2+^]_e_; Sims and Hartell, 2005; Valera et al., 2012; Schmidt et al., 2013; Ishiyama et al., 2014; Baur et al., 2015). The number of release sites per synapse is small and has been estimated by amplitude fluctuation analysis of excitatory postsynaptic currents (EPSCs) to be on average in the range of ~2–5, perhaps with some target- or species-dependent differences (Schmidt et al., 2013; Ishiyama et al., 2014; Malagon et al., 2016). In electron microscopy ~8 docked vesicles were found in PF terminals (Xu-Friedman et al., 2001). These results indicate that single PF AZs harbor more than one release site, consistent with multi-vesicular release (Crowley et al., 2007).

### High-Frequency Facilitation and Low-Frequency Depression

**Figure 1:**
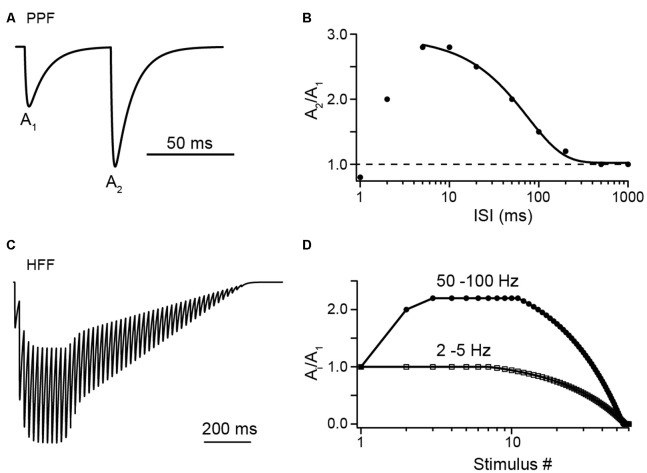
Illustrations of facilitation vs. depression at Parallel fiber (PF) to Purkinje cell (PC) synapses with synthetic data. **(A)** A pair of excitatory postsynaptic currents (EPSCs), normalized to the first amplitude (A_1_), as would be evoked by PF tract stimulation at an interstimulus interval (ISI) of 50 ms, illustrating paired-pulse facilitation (PPF). **(B)** Illustration of paired-pulse ratio (PPR) as a function of ISI. Synthetic data in the range of 5 ms to 1 s were fit by a sigmoidal function (see Valera et al., 2012). Note the decline in PPR at ISI <5 ms, which may indicate a limit in the speed of synaptic vesicle (SV) replenishment (see text for detail). **(C)** Illustration of high-frequency facilitation (HFF) of normalized EPSCs during a 50 Hz train of action potentials (APs). **(D)** Illustration of HFF (circles) and low-frequency depression (LFD; squares) as a function of stimulus number as would be observed at the indicated frequencies (see Doussau et al., 2017).

PF synapses show paired-pulse facilitation (PPF) with paired-pulse ratios (PPRs) between the first and the second EPSC amplitude (A_2_/A_1_) of ~2–3 at small interstimulus intervals (ISIs) of 5–10 ms (Figure 1). PPRs (A_i_/A_1_) remain at this level even during longer lasting high-frequency bursts and under conditions of elevated initial *p*_v_ (*p*_v1_; Atluri and Regehr, 1996; Sims and Hartell, 2005; Valera et al., 2012; Ishiyama et al., 2014; Brachtendorf et al., 2015; Turecek and Regehr, 2018). In light of the above brief overview of biophysical characteristics, this is surprising at first glance. Assuming *N* of three, *p*_v1_ of 0.25 and *p*_v2_ of 0.84 as estimated for PF to PC synapses in 2 mM [Ca^2+^]_e_ (Valera et al., 2012; Schmidt et al., 2013; Brachtendorf et al., 2015), the theoretical maximum for the PPR between second and first pulse in the absence of SV replenishment [PPR = (*p*_v2_/*p*_v1_)*(1 − *p*_v1_) = 2.52; the term (1 − *p*_v1_) accounts for the reduction in *N*_occ_ during the first AP] is close to or even lower than the experimentally found values and subsequent pulses cannot be explained. Consistently, it has been suggested early that SV replenishment at PF terminals is very rapid (Crowley et al., 2007).

Rapid replenishment alone, however, is unlikely to fully account for PPF at PF synapses. It was recognized that even if *p*_v2_ of one and full replenishment between APs (i.e., *N*_occ,1_ = *N*_occ,2_; PPR = *p*_v2_/*p*_v1_) are assumed the experimentally determined values frequently exceed the theoretical maxima (Valera et al., 2012; Ishiyama et al., 2014; Brachtendorf et al., 2015; Miki et al., 2016). Consistently, Valera et al. (2012) found evidence for changes in *N* during activity of PF to PC synapses. They found that *N*, as estimated by the binominal parameter in fluctuation analysis, increased during high-frequency trains of APs. In particular *N* during the second AP was larger than during the first AP (*N*_2_ > *N*_1_), suggesting incremental *N* as a substantial factor of PPF. These findings were subsequently confirmed by stationary fluctuation analysis at single PF to PC synapses in paired recordings (Brachtendorf et al., 2015).

In the latter study, it was proposed that the PPR of PF to PC synapses can be explained by a model with sequential SV pools originally proposed for crayfish motoneuron synapses (Millar et al., 2005). In the adaptation for the PF terminal, it was assumed that release sites are restored from replenishment sites in a Ca^2+^ dependent manner (Brachtendorf et al., 2015). The model well predicted the experimental PPF over a broad range of ISIs of 5 ms to 1 s if a transient increase in *N* between the two APs of a paired-pulse experiment was permitted rather than an increase in *p*_v_ alone. Morphologically, additional *N* appear possible since the area of the PF AZ (0.068 μm^2^; Kusch et al., 2018) is sufficiently large to harbor more than 2–8 release-ready SVs (*r* = 21 nm; Wilhelm et al., 2014).

**Figure 2:**
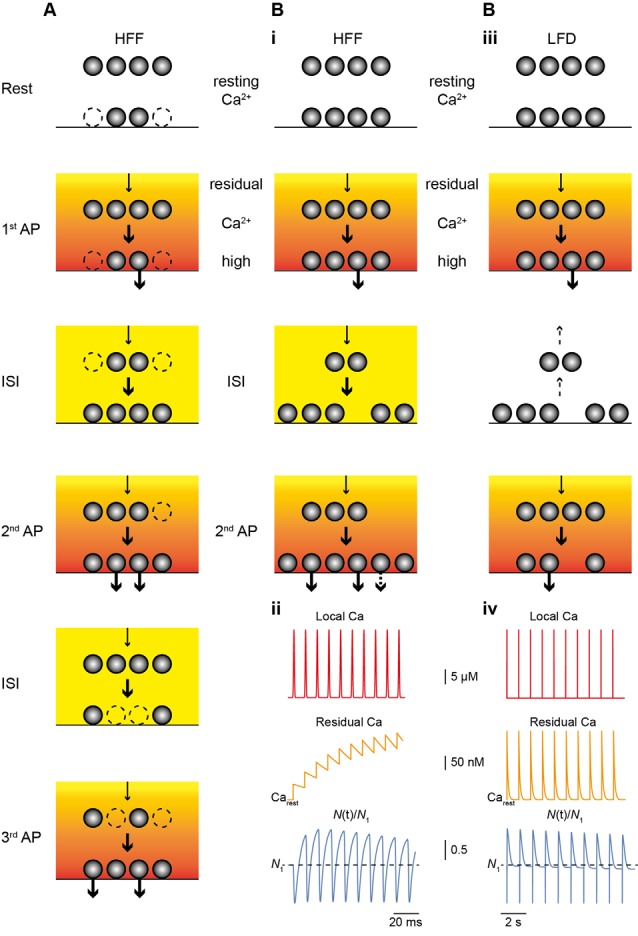
Main mechanism of facilitation and depression. **(A)** Scheme of the model of HFF proposed by Miki et al. (2016). The resting occupancy of release sites (lower vesicles) is ~50% (dashed vesicles; *N*_occ,1_ < *N*), while a replenishment pool (upper vesicles) is fully occupied. The reserve pool is not shown. During high-frequency stimulation *N*_occ_ increases (*N*_occ,train_ = *N*), thereby, giving rise to HFF. Release and the transition of vesicles from replenishment sites to release sites (arrows) is driven by the Ca^2+^ gradient (red to yellow gradient) and residual Ca^2+^ (orange to yellow). **(B)** As in **(A)** but for the model proposed by Doussau et al. (2017) for HFF and LFD. At rest, all release sites are occupied (*N*_occ,1_ = *N_1_*). (i) During HFF, *N* increases (*N*_train_ > *N*_1_); an increase in *p*_v_ makes a smaller contribution to HFF (indicated by the dashed arrow in the lower panel). (ii) Illustrations of local Ca^2+^ at the release sensor (red, *top*), build-up of residual Ca^2+^ (orange, *middle*), and *N(*t) (blue, *bottom*), normalized to *N*_1_ (dashed line), plotted over time for 10 APs at 100 Hz. Note the increase in *N*. (iii) During low-frequency activation Ca^2+^ drops back to resting level between pulses and vesicles return from release sites to replenishment sites (dashed arrows), giving rise to PPR of ~1 in the second pulse. (iv) As in (ii) but for 10 APs at 2 Hz. Note that there is no build-up in residual Ca^2+^ (*middle*) and that *N* is no longer increased at the time of stimulation and progressively declines (*lower*), resulting in LFD during continuing activation.

Two recent studies investigated the mechanisms of sustained release reliability at PF terminals during trains of APs in great depth (Miki et al., 2016; Doussau et al., 2017; Figure 2). Miki et al. (2016) challenged PF to MLI synapses with trains of eight APs delivered at small ISI of 5 ms in elevated [Ca^2+^]_e_ of 3 mM. Based on these data they suggest a sequential two-pool model (plus an implicit reserve pool) that explains facilitation mainly based on increasing *N*_occ_ (Figure 2A). They suggest an initially incomplete resting occupancy of release sites (referred to as docking sites), such that *N*_occ,1_ < *N*_1_. *N*_1_ was estimated to be ~4–5 with a resting occupancy of 0.45, such that *N*_occ,1_ is ~2–3. Replenishment sites of about the same number (4–5) were considered to be fully occupied and the transition probability between the two pools was estimated to be 0.6 during activity. Based on EGTA effects, this high transition probability was Ca^2+^ dependent and gave rise to the increase in *N*_occ_ during the train.

Doussau et al. (2017) challenged PF synapses by long-lasting trains of 50 to >100 APs delivered either at high (ISI 10 or 20 ms) or low (ISI 0.2, 0.5 or 2 s) frequency. They found sustained high-frequency facilitation (HFF) for a large number of 20–30 APs before synapses progressively depressed and frequently became “silent.” Remarkably, during low-frequency activation, synapses no longer facilitated but had A_i_/A_1_ PPRs of 1 for the first ~7 APs. Subsequently, EPSC amplitudes progressively depressed over tens of APs and eventually the synapses became silent. Hence, PF terminals show HFF but low-frequency depression (LFD; Figures 1C,D). In agreement with the above studies, the authors provide evidence that these bidirectional short-term plasticity characteristics are explained by the presence of two sequential SV pools (termed fully releasable and reluctant pool, plus an implicit reserve pool). During high-frequency trains, *N*, which is equal to *N*_occ_ in this study, increased *via* rapid recruitment from the reluctant pool while release sites became progressively depleted during low-frequency stimulation. The results with EGTA and simulations indicated that this rapid recruitment is Ca^2+^ dependent and slowly reversible within ~200 ms, such that it effectively increased *N* contributing to release with high-frequency but not low-frequency AP firing (Figure 2B).

In summary, while there is some controversy about the resting occupancy of release sites (Miki et al., 2016; Doussau et al., 2017), several lines of evidence from the recent literature suggest that facilitation at PF synapses mainly results from a presynaptic mechanism that increases the number of release sites or their occupancy during high-frequency trains of APs. This increase is the result of a very rapid, activity-dependent supply of SVs from replenishment sites, also referred to a “overfilling” of the ready releasable pool (RRP; Neher and Brose, 2018). The forward transition of SVs from replenishment sites is likely to be reversible on a slower time-scale, thereby, explaining the finding of LFD in addition to HFF at PF synapses.

### Mechanisms of Rapid Replenishment

The very rapid, Ca^2+^ dependent forward transition of SVs from replenishment sites to release sites requires a mechanism that operates on the ms time-scale. PPR experiments indicate that the speed of the replenishment process reaches its limit at ISI <5 ms (Figure 1B). At shorter ISI PPF declined and eventually turned to depression (Valera et al., 2012).

Assuming an exponential process, Miki et al. (2016) estimate a very rapid rate constant of ~180 s^−1^, corresponding to τ of ~5.5 ms per release site for an ISI of 5 ms. This is faster than would be obtained by mere diffusion of SVs, suggesting an active process. Consistently, they found evidence for an involvement of actin and myosin cytoskeleton in rapid replenishment based on the inhibitory effects of latrunculin B and blebbistatin. Additional experiments with EGTA-AM revealed the Ca^2+^ dependency of replenishment.

As detailed above, Doussau et al. (2017) found evidence that the replenishment process is reversible on a slower time scale of ~200 ms. Interestingly, in a recent manuscript reporting results from electron microscopic analysis of hippocampal synapses, following stimulation and rapid freezing of cultured neurons, new SVs were recruited to the plasma membrane and fully replenished the docked pool of SVs within ~10 ms after stimulation (Kusick et al., 2018). The docking of these SVs was transient and they either undocked or fused within 100 ms. These ultra structural results are in notable agreement with the findings at PF synapses, suggesting that recruitment of SVs to release sites is rapid and reversible.

Already 20 years ago it has been suggested that facilitation at PF synapses requires a Ca^2+^ dependent facilitation sensor separate from the release sensor (Atluri and Regehr, 1996). The molecular identity and mode of action of this sensor, however, remained elusive until recently. Recent results suggest that Synaptotagmin 7 (Syt7) acts as facilitation sensor at PF terminals. Syt7 knock-out mice displayed reduced PPF, while their *p*_v_ and presynaptic Ca^2+^ signaling were not affected (Turecek and Regehr, 2018). Mechanistically, Ca^2+^ binding to the C2A domain of Syt7 is required for facilitation at different synapses (Jackman et al., 2016). Interestingly, Syt7 was also found to promote SV replenishment during trains of APs in a Ca^2+^ dependent manner by interaction with Ca^2+^ bound calmodulin (Liu et al., 2014). For other functions of Syt7, e.g., in asynchronous release (Turecek and Regehr, 2018), and proposed relationships between different functions I refer the reader to recent reviews (e.g., Chen and Jonas, 2017; Bornschein and Schmidt, 2019; Volynski and Krishnakumar, 2018).

At Syt7 mutant PF synapses, a substantial amount of PPF remained at short ISI (Turecek and Regehr, 2018). This indicates that other mechanisms are operational in addition, which may involve other proteins with C2 domains such as Munc13s (Neher and Brose, 2018). The cerebellum-specific Munc13-3, for example, increases *p*_v_ and alters PPR by “superpriming” (Augustin et al., 2001; Ishiyama et al., 2014). Experiments in a developmental context indicated that Munc13-3 tightens the coupling distance between SVs and P/Q-type channels (Kusch et al., 2018). Whether coupling distance tightening and Munc13-3 can establish newly occupied release sites during high-frequency activity is unclear at present. For further details on molecular mechanisms of short-term plasticity and the role of Synaptotagmins and other molecular players I refer the reader to recent comprehensive reviews (Jackman and Regehr, 2017; Bornschein and Schmidt, 2019; Neher and Brose, 2018; Volynski and Krishnakumar, 2018).

## Concluding Remarks

PPF was discovered more than 70 years ago and its mechanisms may differ between synapses (Jackman and Regehr, 2017). At different synapses different conceptions were suggested to account for facilitation. Originally, it has been proposed that the “active Ca^2+^,” which is “Ca^2+^ remaining attached to specific sites on the inner axon membrane” causes facilitation (Katz and Miledi, 1968). Reminiscent of the active Ca^2+^ are slow Ca^2+^ unbinding from the release sensor (Bornschein et al., 2013) and Ca^2+^ binding to the facilitation sensor Syt7 (Atluri and Regehr, 1996; Jackman et al., 2016). In addition, elevated release site [Ca^2+^]_i_ due to AP broadening (Geiger and Jonas, 2000) or effects of endogenous Ca^2+^ buffers can cause facilitation (Rozov et al., 2001). Finally, the very rapid, activity-dependent increase in the number of occupied release sites added to the mechanisms of facilitation (Valera et al., 2012; Brachtendorf et al., 2015; Miki et al., 2016; Doussau et al., 2017). At PF synapses buffering by their major endogenous buffer Calretinin increases PPF by reducing *p*_v1_ (Schmidt et al., 2013; Brachtendorf et al., 2015). The effect is attenuated by the concomitant reduction in the Ca^2+^ dependent recruitment process such that the net effect of Calretinin on PPF is rather moderate (Schiffmann et al., 1999; Brachtendorf et al., 2015). Also, Ca^2+^ unbinding from the release sensor likely makes a small contribution (Brachtendorf et al., 2015; Doussau et al., 2017). The majority of facilitation, however, results from an ultra-rapid and reversible increase in occupied release sites during high-frequency activity (Miki et al., 2016; Doussau et al., 2017). Hence, release sites at AZs of PF synapses are very dynamic entities that can be reversibly recruited or replenished on a millisecond time scale.

## Author Contributions

HS wrote the manuscript.

## Conflict of Interest Statement

The author declares that the research was conducted in the absence of any commercial or financial relationships that could be construed as a potential conflict of interest.

## Abbreviations

A: amplitude of EPSC
AP: action potential
AZ: active zone
BC: Basket cell
EPSC: excitatory postsynaptic current
MLI: molecular layer interneuron
*N*: number of release sites
*N*_occ_: number of release sites occupied by release-ready SVs
*p*_v_: vesicular release probability
SV: synaptic vesicle
PC: Purkinje cell
PF: parallel fiber
PPR: paired pulse ratio
PPF: paired pulse facilitation
RRP: ready releasable pool.
